# Photosynthesis of co-existing *Phragmites* haplotypes in their non-native range: are characteristics determined by adaptations derived from their native origin?

**DOI:** 10.1093/aobpla/plt016

**Published:** 2013-02-27

**Authors:** Loc Xuan Nguyen, Carla Lambertini, Brian K. Sorrell, Franziska Eller, Luciana Achenbach, Hans Brix

**Affiliations:** Department of BioScience, Plant Biology, Aarhus University, Ole Worms Allé 1, DK-8000 Aarhus C, Denmark

**Keywords:** Adaptations, Gulf Coast of North America, genotypes, haplotypes, invasion, photosynthesis, *Phragmites*

## Abstract

The Gulf Coast of North America (GC) is a ‘hot spot’ of *Phragmites* diversity as several lineages (defined according to the haplotypes of their chloroplast DNA) differing in origin, genetic traits and phenotype co-exist and interbreed in this area. We analysed differences in photosynthetic characteristics among and within four haplotypes to understand if differences in gas exchange can be attributed to adaptations acquired in their native ranges. We collected rhizomes of four GC haplotypes (I2, M1, M and AI; including the phenotypes ‘Land-type’, ‘Delta-type’, ‘EU-type’ and ‘Greeny-type’) and propagated them in a common controlled environment to compare photosynthesis–irradiance responses, CO_2_ responses, chlorophyll fluorescence, the activity of ribulose-1,5-bisphosphate carboxylase/oxygenase (Rubisco), specific leaf area (SLA), pigment contents, stomatal density and guard cell length. The maximum light-saturated photosynthetic rate, *A*_max_, in the haplotype I2 (Land-type) and haplotype M1 (Delta-type) (34.3–36.1 µmol CO_2_ m^−2^ s^−1^) was higher than that in the invasive Eurasian haplotype M (22.4 ± 2.3 µmol CO_2_ m^−2^ s^−1^). The *A*_max_ of haplotype AI (Greeny3-type) was 29.1 ± 4.0 µmol CO_2_ m^−2^ s^−1^ and did not differ from the *A*_max_ of the other haplotypes. The carboxylation rate (*V*_cmax_) and electron transport rate (*J*_max_) followed the same pattern as *A*_max_. The haplotypes also differed in SLA (17.0–24.3 m^2^ kg^−1^ dry mass) and pigment content, whereas stomatal density and guard cell length, Rubisco activity and chlorophyll fluorescence did not differ significantly among haplotypes. The high photosynthetic activity and gas-exchange capacity of the two haplotypes originating in tropical Africa and the Mediterranean area (haplotypes I2 and M1) are apparently adaptations derived from their native ranges. Hence, the haplotypes can be regarded as ecotypes. However, it remains unclear how these differences relate to plant competitiveness and fitness in the GC of North America environment.

## Abstract (precis)

Several Phragmites lineages differing in origin and phenotype co-exist in the Gulf Coast of North America. We collected rhizomes of four lineages and propagated them in a common environment to compare photosynthetic characteristics. We observed substantial differences among and within lineages. As the lineages originating in Africa and in the Mediterranean region had higher photosynthetic capacity than the lineages originating in Eurasia, and showed typical ecophysiological traits of plants adapted to warm and arid climates, we concluded that the differences observed are due to adaptations acquired in the native ranges. The four lineages can therefore be regarded as ecotypes.

## Introduction

The common reed, *Phragmites australis* (Cav.) Trin. ex Steud, is a cosmopolitan emergent wetland grass occurring on all continents except Antarctica and in subtropical to cold temperate climates (Brix 1999; Clevering and Lissner 1999). It grows well in wet habitats, along rivers and in the littoral zone of lakes down to ca. 3 m water depth due to its ability to aerate rhizomes and roots by efficient convective gas through-flow (Armstrong and Armstrong 1991; Brix *et al.* 1992; Armstrong *et al.* 1999). There is, however, considerable morphological variability between different populations and genotypes of *P. australis*, some of which may be explained by the large geographic range of the species and different ploidy levels (Clevering and Lissner 1999). The morphological, cytological and geographical variation within the species accompanies substantial differences in ecophysiology and clonal development (Rolletschek *et al.* 1999; Kühl and Zemlin 2000; Mozdzer and Zieman 2010; Achenbach *et al.* 2012; Eller and Brix 2012). Also, different genotypes of *P. australis* possess different degrees of phenotypic plasticity in photosynthesis when grown under common garden environments in different climates (Lessmann *et al.* 2001; Eller and Brix 2012).

In the Mississippi River Delta at the Gulf Coast of North America, five maternal lineages of *Phragmites*, defined according to their chloroplast DNA haplotypes (Saltonstall 2002) and hereafter termed haplotypes, have been identified (Hauber *et al.* 2011; Lambertini *et al.* 2012*a*). These haplotypes differ in their geographic origin and to a large extent in their phenotype. Nevertheless, there is no complete match between haplotype and phenotype, as one of the phenotypes belongs to three different haplotypes. This variation pattern is probably due to gene flow among haplotypes in the Gulf Coast, as evidenced by nuclear DNA variation (Lambertini *et al.* 2012*a*).

The Land phenotype (or Land-type) is defined by its red, woody, branched stems and is prevalent inland from Texas to Florida, with only scattered occurrences in the Mississippi River Delta (Lambertini *et al.* 2012*a*). It is a hybrid between the species *Phragmites mauritianus* and *P. australis*, probably formed in tropical Africa where the two species co-occur. The Land-type has haplotype I2 like *P. mauritianus* in Uganda and Burkina Faso (Lambertini *et al.* 2012*a*).

The Delta-type is the predominant phenotype in the delta and is identified by its tall shoots. It is an introduction from the populations of the Mediterranean region (Southern Europe, North Africa and the Middle East; Lambertini *et al.* 2012*a*) with which it shares haplotype M1.

The EU-type is identified by its purple inflorescence and small size compared with the Land- and Delta-types (also named ‘short forms’ by Hauber *et al.* 2011). It is a relatively recent arrival of the North American population of the invasive Eurasian *P. australis* with haplotype M which has spread in coastal wetlands along the Atlantic coast (Lambertini *et al.* 2012*a*).

The Greeny-type occurs in small stands scattered in the Mississippi River Delta. The name refers to its characteristic blue-green stand colour. The Greeny phenotype belongs to three different haplotypes, namely haplotype M (hence Greeny1-type), haplotype AD (hence Greeny2-type) and haplotype AI (hence Greeny3-type). All Greeny haplotypes have been found in Europe (Lambertini *et al.* 2012*b*), but one of them, the Greeny3-type, may be native to South Africa and introduced to the Mississippi River Delta from Europe.

Despite the presence of haplotype M, the genetic pattern of the *P. australis* population at the Gulf Coast is very different from that of the East Coast (Lambertini *et al.* 2012*a*) where 14 closely related native American haplotypes and one distantly related Eurasian invasive haplotype (haplotype M) co-exist (Saltonstall 2002). In the Gulf Coast, three Eurasian haplotypes co-occur with one Mediterranean and one tropical African haplotype (Lambertini *et al.* 2012*a*). The European and Mediterranean haplotypes are closely related to each other and distantly related to the tropical African haplotype. In addition, haplotype M shows two distinct phenotypes in the Gulf Coast: the EU-type and the Greeny-type. While the EU-type is exclusive to haplotype M, the Greeny-type is shared by all European-related haplotypes (M, AD and AI). None of these haplotypes appears to be native to the Gulf Coast; haplotype I2 with Land-type phenotype has been present in the Gulf Coast for the longest time (Lambertini *et al.* 2012*a*), whereas the introduction of haplotypes M, M1, AD and AI probably occurred in the last couple of centuries (Chambers *et al.* 1999; Saltonstall 2002; Lambertini *et al.* 2012*a*).

The aim of this study was to assess whether *Phragmites* haplotypes co-occurring in the Mississippi River Delta differ in their photosynthetic characteristics and whether these differences are shared by all genotypes of the same haplotype, hence being attributable to ecotypes, or alternatively have resulted after colonization of the delta. We grew one to four genotypes of four haplotypes in a common controlled environment to ensure that possible differences in photosynthetic characteristics were due to genetic differences and were not affected by the variable environmental conditions in the delta. Achenbach *et al.* (2012) showed that ecophysiological traits of *P. australis* are not related to geographic range, but are genotype dependent. We therefore analysed intra-haplotypic variation in photosynthetic characteristics in order to evaluate whether differences among haplotypes are consistent among genotypes within each haplotype or whether genotypes deviating in photosynthetic characteristics from the other genotypes of the same haplotype could be recognized by their phenotype (e.g. EU-type vs. Greeny1-type within haplotype M).

## Methods

### Plant material

Eleven genotypes of *P. australis* were collected in June 2009 from visually distinct stands in the Mississippi River Delta marshes (Table 1). Rhizomes from the genotypes were transferred to a greenhouse at Aarhus University, Denmark (56°13′N; 10°07′E) and grown in 80-L tanks containing a mixture of sand and commercial compost. DNA was extracted from the genotypes for genetic analysis (Lambertini *et al.* 2012*a*). Haplotypes were previously determined by Lambertini *et al.* (2012*a*) based on chloroplast DNA sequences (*trn*T-*trn*L and *rbc*L-*psa*I regions) by following the classification systems introduced by Saltonstall (2002). Phenotypes were determined based on nuclear DNA variation (microsatellites, amplified fragment length polymorphisms and sequences), which matched the overall appearance of the stands in the field (Lambertini *et al.* 2012*a*). The genotypes were identified as ‘haplotype I2, Land-type’ (three genotypes), ‘haplotype M1, Delta-type’ (four genotypes), ‘haplotype M, EU-type’ (two genotypes), ‘haplotype M, Greeny1-type’ (one genotype) and ‘haplotype AI, Greeny3-type’ (one genotype).

**Table 1:** List of *Phragmites* genotypes used in the study.

Genotype^a^	Haplotype^b^	GenBank accession no.	Phenotype^c^	Distribution and origin^c^
ROMS3	I2	HQ664450 + AY016334	Land-type	Distributed along the Gulf Coast of North America from Texas to California and scattered in the Mississippi Delta; originating in tropical Africa and anciently established in America
ROM16	I2	HQ664450 + AY016334	Land-type	
WHS3	I2	HQ664450 + AY016334	Land-type	
ROMS4	M1	JF271678 + AY016335	Delta-type	Dominant phenotype in the Mississippi River Delta; originating from the Mediterranean region (North Africa, Middle East, Southern Europe)
ROMS7^d^	M1	JF271678 + AY016335	Delta-type	
WHS4	M1	JF271678 + AY016335	Delta-type	
ROM4	M1	JF271678 + AY016335	Delta-type	
ROM2	M	AY016327 + AY016335	EU-type	Distributed throughout North America; distinguished from Land and Delta phenotype by its purple panicles and smaller size; introduced from Eurasia
WHS2	M	AY016327 + AY016335	EU-type	
OCT1	M	AY016327 + AY016335	Greeny1-type	
Pa107gcUS	AI	AY016326 + HQ664451	Greeny3-type	Exclusively found in the Mississippi Delta; can be distinguished by its bright blue-green leaves, introduced from Europe

^a^Labels according to Hauber *et al.* (2011). ROM, Romere Pass and its distributaries; ROMS, south of Romere Pass; WHS, White Splay; OCT, Octave Pass.

^b^Haplotype I2 is the Gulf-Coast lineage (Saltonstall *et al.* 2004) and the cp-microsatellite variant I2 of haplotype I (Lambertini *et al.* 2012*b*).

^c^According to Lambertini *et al.* (2012*a*).

^d^Genotype ROMS7 (Delta-type) shares alleles with the EU-type genotypes. All genotypes are from the interior marshes of the Mississippi Delta. All the rhizomes were collected in June 2009.

Rhizomes were transplanted to 6-L plastic pots containing a mixture of quartz sand and commercial compost, mostly consisting of *Sphagnum* (Pindstrup No. 2, Pindstrup Mosebrug, Ryomgaard, Denmark). The pots were placed in a growth chamber (Bio 2000S, Weiss Umwelttechnik, Lindenstruth, Germany) under a day/night cycle of 14/10 h, a temperature of 25/22 °C and a relative air humidity of 70/80 %. Light was provided by metal halide lamps at a photosynthetic photon flux density (PPFD) of ∼150 µmol m^−2^ s^−1^ at the base and 500–600 µmol m^−2^ s^−1^ at the top of the plants. Each potted plant was placed in its own outer container to keep the soil water saturated. Plants were watered three times per week with a commercial fertilizer solution (Pioner NPK Makro 10-4-25 + Mg and Pioner Mikro + Fe, Brøste, Denmark). The genotypes were allowed to acclimate to the growth conditions in the growth chamber for 10 weeks before ecophysiological measurements were initiated.

### Photosynthetic light response

The youngest fully developed leaves (the third or the fourth from the apex) of three shoots from each genotype were used for the photosynthetic gas exchange measurements. Measurements were made with a Li-Cor 6400XT Portable Photosynthesis System (Li-Cor, Nebraska, USA) equipped with a Li-Cor 6400-02B LED light source. The sample chamber temperature was controlled at 25 °C and the relative air humidity at 35–50 % for all measurements. Photosynthetic light responses were determined from measurements at nine irradiances (2000, 1500, 1000, 700, 500, 250, 120, 60 and 30 µmol m^−2^ s^−1^) at 400 ppm CO_2_ in the sample chamber. Leaves were acclimated in the leaf chamber for 3–5 min until steady-state gas exchange was achieved, and data then recorded using the light curve program of the Li-Cor IRGA. The light-response curve of each leaf was fitted using the quadratic equation of Prioul and Chartier (1977):
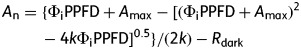



where *A*_n_ (µmol CO_2_ m^−2^ s^−1^) is the net assimilation rate; Φ_i_ (mol CO_2_ (mol photons)^−1^) is the initial slope of the light-response curve or apparent quantum yield; PPFD is the irradiance (µmol m^−2^ s^−1^); *A*_max_ (µmol CO_2_ m^−2^ s^−1^) is the light-saturated rate of gross photosynthesis; *k* is the convexity; and *R*_dark_ (µmol CO_2_ m^−2^ s^−1^) is the dark respiration rate. The dark respiration (*R*_dark_), the light compensation point (*I*_c_), the light saturation point (*I*_k_), and Φ_i_ and *A*_max_ were estimated from individual light-response curves using Photosyn Assistant software, version 1.1.2 (Dundee Scientific, Dundee, UK). The *A*_n_ at 0 PPFD was inferred from the above equation. The data from the light-response curves at light saturation were used to calculate the intrinsic water-use efficiency (WUE_i_) as the ratio of *A*_max_ to stomatal conductance (*g*_s_), also measured by the Li-Cor 6400XT.

### Photosynthetic CO_2_ response

The leaves used for the light-response curves were marked and also used for CO_2_-response curves. The CO_2_-response curves were taken at nine CO_2_ concentrations (800, 700, 600, 500, 400, 300, 200, 100 and 50 ppm). CO_2_ was supplied with a 12-g CO_2_ cartridge mounted in the Li-Cor 6400XT and assimilation was measured at a PPFD of 2000 µmol m^−2^ s^−1^ and at 25 °C.

The carbon fixation kinetic models for terrestrial C3 plants (Farquhar *et al.* 1980) as modified by Dubois *et al.* (2007) and Sharkey *et al.* (2007) were used to describe the relationship between the net carbon assimilation rate, *A* (µmol CO_2_ m^−2^ s^−1^), and the intercellular CO_2_ concentration, *C*_i_ (µmol mol^−1^), and to estimate the key biochemical limitations to steady-state C3 photosynthesis:
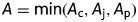


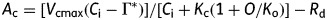


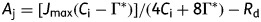


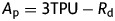



In these equations, *A*_c_, *A*_j_ and *A*_p_ are the net CO_2_ assimilation rates (µmol CO_2_ m^−2^ s^−1^) limited by Rubisco, ribulose-1,5-bisphosphate (RuBP) regeneration and triose phosphate use (TPU), respectively. *V*_cmax_ is the maximum carboxylation rate of Rubisco (µmol m^−2^ s^−1^); *R*_d_ is the rate of mitochondrial respiration (µmol CO_2_ m^−2^ s^−1^); *K*_c_ and *K*_o_ are the Rubisco Michaelis–Menten constants for CO_2_ (µmol mol^−1^) and O_2_ (mmol mol^−1^), respectively; *O* is the partial pressure of oxygen (mmol mol^−1^); *J*_max_ is the maximum rate of electron transport to reduce NADP^+^ for RuBP regeneration (µmol e^−^ m^−2^ s^−1^); and Γ* is the photorespiratory CO_2_ compensation point (µmol mol^−1^). The Michaelis–Menten constants (*K*_c_ = 406.8 µmol mol^−1^, *K*_o_ = 275.7 mmol mol^−1^) and the photorespiratory CO_2_ compensation point (Γ* = 28.7 µmol mol^−1^) were adjusted using the temperature coefficients from Bernacchi *et al.* (2003) and Dubois *et al.* (2007).

Carbon dioxide assimilation was assumed to be Rubisco limited at *C*_i_ < 250 µmol mol^−1^; hence, the *A*/*C*_i_ datasets with *C*_i_ < 250 µmol mol^−1^ were fitted to *A*_c_ to estimate *V*_cmax_ and *R*_d_ (Dubois *et al.* 2007). Then *R*_d_ estimated from *A*_c_ was used to estimate *J*_max_ from *A*_j_ using *A*/*C*_i_ datasets with *C*_i_ > 250 µmol CO_2_ mol^−1^. Our data did not allow estimation of TPU.

### Stomatal density and length

Stomatal density (mm^−2^) and the length of guard cells (µm) were determined on the third or fourth youngest leaf. Three new leaves produced during the 10-week experiment in the growth chamber were harvested from each genotype and fixed in 70 % ethanol for 7 days and then hydrated by placing them in 70, 50, 25 and 0 % ethanol sequentially, for at least 15 min in each solution. The leaves were cleared in fuchsin-KOH for 24 h at 60 °C, then rinsed in water and dehydrated by placing the leaves in water first and then sequentially in 70, 96 and 99 % ethanol. Finally, leaves were embedded in Euparal (Carl Roth Gmbh, Karlsruhe, Germany). Stomatal density and the length of guard cells on both upper (adaxial) and lower (abaxial) sides of leaves were determined under ×40 magnification with a calibrated reticule in a light-transmission microscope.

### Specific leaf area and pigment analysis

After taking photosynthetic light and CO_2_ response measurements, the leaves were cut and their one-sided area was obtained by the weight/area ratio of photocopies of the leaves. The leaves were then lyophilized in a freeze-drier for the determination of dry mass (DM). The specific leaf area (SLA, m^2^ kg^−1^ DM) was calculated as leaf area per dry mass. Pigments (mg g^−1^ DM) were extracted in 96 % ethanol and the contents of chl*_a_*, chl*_b_*, chl*_a_*_+*b*_ and total carotenes and xanthophylls (total carotenoids) were analysed spectrophotometrically according to Lichtenthaler (1987). Pigments (mg m^−2^) were obtained as the ratio between pigments (mg g^−1^ DM) and SLA (m^2^ kg^−1^ DM) obtained as described above.

### Rubisco activity

Three leaves of each genotype were harvested for the analysis of initial Rubisco activity (non-activated Rubisco) and total Rubisco activity following Hansen *et al.* (2007). Harvesting took place in the light and the entire leaf was immediately frozen in liquid nitrogen after excision. The frozen leaf was ground in a mortar containing liquid nitrogen. Approximately half a laboratory spoon of the ground leaf material was then transferred to a chilled mortar and further ground in 5 mL of extraction buffer containing 50 mM Bicine (pH 8), 1 mM EDTA-Na_2_, 10 mM MgCl_2_, 5 mM dithiothreitol (DTT), 10 mM isoascorbate and 2 % (w/v) polyvinylpyrrolidone. Initial Rubisco activity was determined in an assay solution consisting of 500 mM Bicine (pH 8), 1 mM EDTA-Na_2_, 100 mM MgCl_2_, 50 mM DTT, 192.5 mM NaH^14^CO_3_ and 5 mM RuBP. The reaction was initiated by adding ground plant extract and stopped after 60 s with 6 M HCl. Total Rubisco activity was analysed by activation in an assay solution for 5 min before addition of the 5 mM RuBP. The reaction was stopped after 60 s with 6 M HCl. Assays were carried out at 25 °C in a total volume of 300 μl using 6-mL vials. Extracted samples from the assay were dried at 60 °C for 24 h and then re-dissolved in two drops of 6 M NaOH and 1.2 mL of ultra-filtered water. The amount of radioactive decay energy was measured in a scintillation counter (Tri-CARB 2100 TR, Packard, Meriden, USA). The concentration of chl*_a_*_+*b*_ in the extract was analysed using 96 % ethanol and used with chl*_a_*_+*b*_ concentrations from pigment analyses to express activities of Rubisco on a leaf surface area basis (μmol C m^−2^ s^−1^). Rubisco activation state (the percentage of Rubisco that was active in the leaves) was calculated as the ratio of initial activity to total activity.

### Chlorophyll fluorescence

The maximum photochemical yield of photosystem II (PSII) in dark-incubated leaves, *F*_v_/*F*_m_, was measured in three dark-acclimated leaves from each genotype with a Portable Chlorophyll Fluorometer (PAM-2000, Walz Mess- und Regeltechnik, Effeltrich, Germany). Leaves were darkened with a leaf clamp for 15 min prior to measurements, and rapid light curves were measured using pre-installed software to estimate the relative efficiency of photon conversion (quantum yield) at low irradiances (Φ_PAM_), the light saturation point (*I*_k,PAM_, µmol m^−2^ s^−1^) and the maximum electron transport rate (ETR_max_, µmol m^−2^ s^−1^). Differently from *J*_max_, also defined as electron transport rate and related to RuBP regeneration, ETR_max_ refers to the electron transport in PSII activated by the light harvested by the chlorophylls.

### Statistics

The software Statgraphics Centurion XVI (Statpoint Technology Inc., VA, USA) was used to analyse the data. Data were tested for normal distribution and variance homogeneity using Levene's test. The differences among the different *P. australis* haplotypes were tested by nested analysis of variance (ANOVA), with genotype nested in haplotype and using Type III sum of squares and the General Linear Model procedure of the Statgraphics software. *Post hoc* comparisons of means were performed using Tukey's honestly significant difference (HSD) procedure at the 0.05 significance level. When necessary, data were log-transformed to approximate normality and secure variance homogeneity, but for clarity all data are presented as untransformed. A principal component analysis (PCA) was carried out to obtain a small number of linear combinations of parameters which vary significantly among haplotypes and genotypes, and account for most of the variability in the data. The components with eigenvalues >1 were extracted, and biplots of the principal components were obtained to illustrate differences among haplotypes and genotypes.

## Results

### Photosynthetic light responses

**Figure 1.**
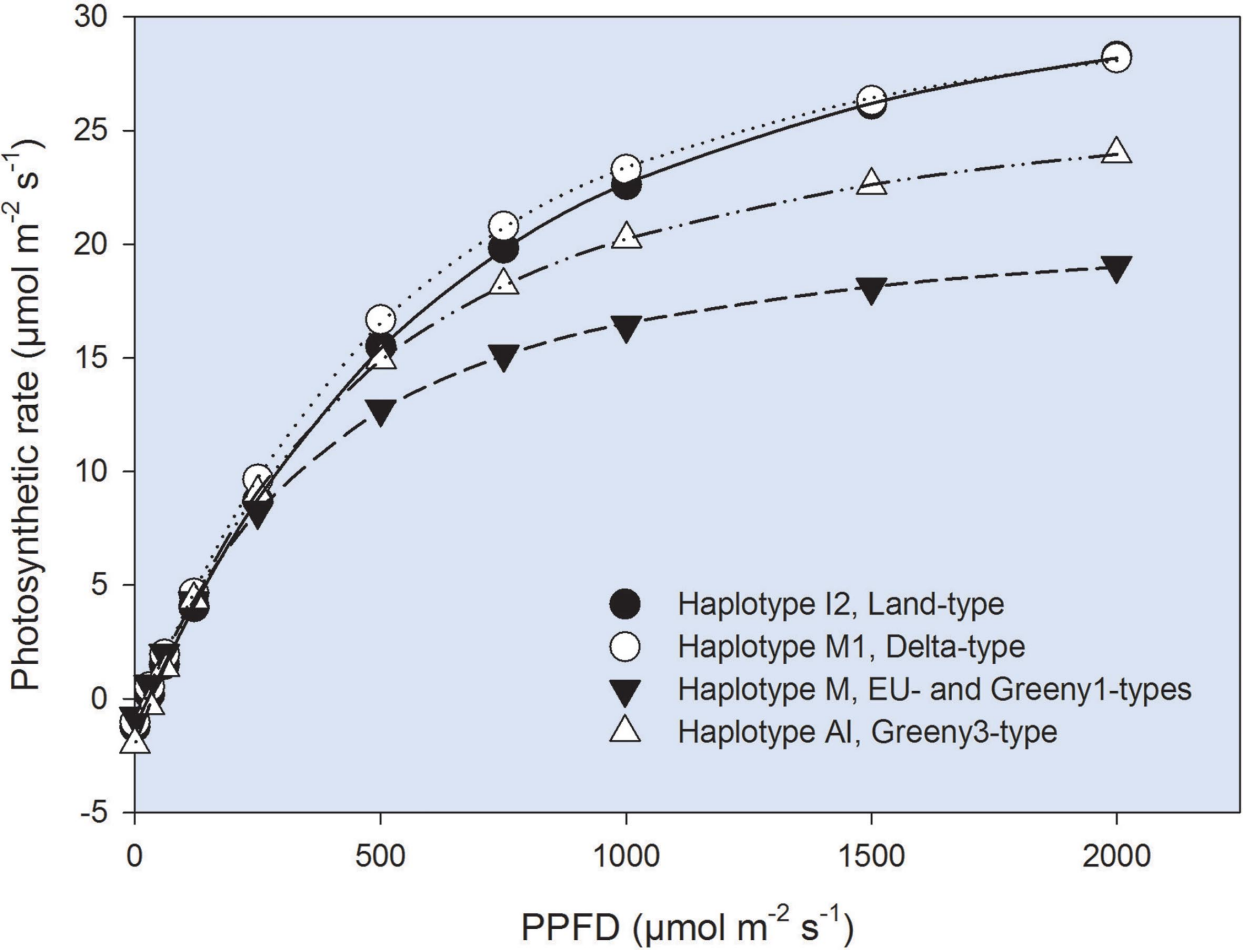
Average photosynthetic light-response curves of four haplotypes of *Phragmites* co-existing at the Gulf Coast of North America. The curves of haplotype I2 (Land-type), haplotype M1 (Delta -type), haplotype M (EU-type and Greeny1-type) and haplotype AI (Greeny3-type) are the average of three, four, three and one genotype, respectively (three light-response curves on three different leaves were analysed for each genotype). All the curves were conducted at 400 ppm CO_2_ and 25 °C.

The light-response curves of the four haplotypes differed significantly for all estimated parameters except the apparent quantum yield, Φ_i_ (Table 2 and Fig. 1). The *A*_max_ and *I*_k_ varied significantly among genotypes within haplotypes. Haplotype M (including the EU-type and Greeny1-type) had consistently lower *A*_max_ and *I*_k_ than haplotype I2 (the Land-type) and M1 (the Delta-type). The *I*_c_ and *R*_dark_ of haplotype M were also lower than those of haplotype I2 but did not differ from haplotype M1. Haplotype AI (the Greeny3-type) did not differ in *A*_max_, *I*_k_, *I*_c_ and *R*_dark_ either from haplotype M or I2 and M1. The WUE_i_ did not differ among the four haplotypes but differed among genotypes within haplotypes (Table 2).

**Table 2:** Photosynthetic characteristics of four haplotypes of *Phragmites* co-existing at the Gulf Coast of North America—light response parameters. Mean (±1 SE) light-saturated rate of photosynthesis (*A*_max_), apparent quantum yield (Φ_i_), light compensation point (*I*_c_), light saturation point (*I*_k_), dark respiration (*R*_dark_) and intrinsic water-use efficiency at maximum light intensity (WUE_i_) and the results (*P* values) of nested two-way ANOVA. Different superscript letters in the columns indicate significant differences (*post hoc* Tukey's HSD test, *P* < 0.05) among haplotypes. Analysis of variance results in bold indicate *P* values <0.05.

Haplotype	*A*_max_ (µmol CO_2_ m^−2^ s^−1^)	Φ_i_ (mol CO_2_ (mol photons)^−1^)	*I*_c_ (µmol m^−2^ s^−1^)	*I*_k_ (µmol m^−2^ s^−1^)	*R*_dark_ (µmol CO_2_ m^−2^ s^−1^)	WUE_i_ (µmol CO_2_ mol^−1^ H_2_O)
I2	36.1 ± 2.3^b^	0.048 ± 0.002	25.8 ± 1.7^b^	784 ± 42^b^	1.23 ± 0.09^b^	40.7 ± 2.3
M1	34.3 ± 2.0^b^	0.052 ± 0.002	19.6 ± 1.5^ab^	683 ± 36^b^	1.02 ± 0.08^ab^	51.6 ± 7.2
M	22.4 ± 2.3^a^	0.049 ± 0.002	15.1 ± 1.7^a^	475 ± 42^a^	0.74 ± 0.09^a^	57.4 ± 6.0
AI	29.1 ± 4.0^ab^	0.051 ± 0.003	27.3 ± 3.0^ab^	602 ± 73^ab^	1.38 ± 0.16^ab^	41.4 ± 11.1
Nested two-way ANOVA results (*P* value)						
Genotype (haplotype)	**0.022**	0.283	0.613	**0.043**	0.589	**0.002**
Haplotype	**0.030**	0.516	**0.022**	**0.014**	**0.036**	0.697

### Photosynthetic CO_2_ responses

**Figure 2.**
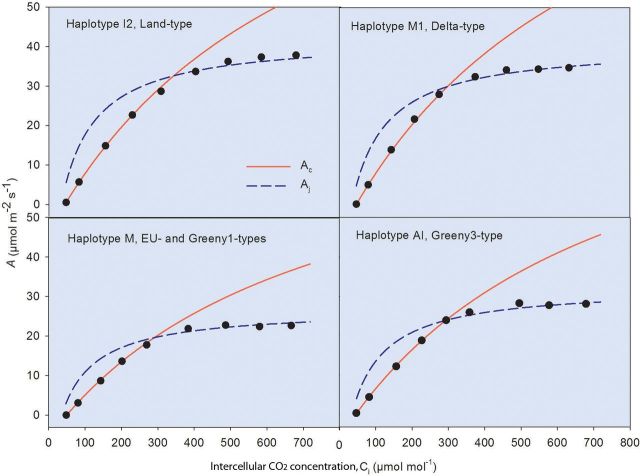
Average CO_2_-response curves of four haplotypes of *Phragmites* co-existing at the Gulf Coast of North America. The curves of haplotype I2 (Land-type), haplotype M1 (Delta-type), haplotype M (EU-type and Greeny1-type) and haplotype AI (Greeny3-type) are the average of three, four, three and one genotype, respectively (three CO_2_-response curves on three different leaves were analysed for each genotype). Assimilation values obtained with intercellular CO_2_ concentration (*C*_i_) <250 (µmol mol^−1^) were fitted to the *A*_c_ curve and assimilation values >250 (µmol mol^−1^) were fitted to the *A*_j_ curve (Dubois *et al.* 2007). All the curves were conducted at a light intensity of 2000 µmol m^−2^ s^−1^ and 25 °C.

The parameters derived from the CO_2_-response curves varied among and within haplotypes (Table 3 and Fig. 2) except for the rate of mitochondrial respiration, *R*_d_, which did not differ among haplotypes. Significant differences were found for *V*_cmax_ and *J*_max_ among haplotypes. The *V*_cmax_ and *J*_max_ were lowest in haplotype M and highest in haplotypes I2 and M1. Values for haplotype AI were intermediate and did not differ from those of haplotypes M and I2 and M1.

**Table 3:** Photosynthetic characteristics of four haplotypes of *Phragmites* co-existing at the Gulf Coast of North America—CO_2_ response parameters. Mean (±1 S.E) mitochondrial respiration (*R*_d_), maximum carboxylation rate (*V*_cmax_) and maximum electron transport rate (*J*_max_) estimated from CO_2_-response curves of individual leaves and the results (*P* values) of nested two-way ANOVA. Different superscript letters in the columns indicate significant differences (*post hoc* Tukey's HSD test, *P* < 0.05) among haplotypes. Analysis of variance results in bold indicate *P* values <0.05.

Haplotype	*R*_d_ (µmol CO_2_ m^−2^ s^−1^)	*V*_cmax_ (µmol m^−2^ s^−1^)	*J*_max_ (µmol m^−2^ s^−1^)
I2	2.37 ± 0.18	118 ± 6^b^	178 ± 10^b^
M1	2.59 ± 0.15	123 ± 6^b^	172 ± 9^b^
M	2.27 ± 0.18	84 ± 6^a^	116 ± 10^a^
AI	1.97 ± 0.31	99 ± 11^ab^	137 ± 17^ab^
Nested two-way ANOVA results (*P* value)			
Genotype (haplotype)	**0.006**	**0.041**	**0.014**
Haplotype	0.411	**0.021**	**0.019**

### SLA and pigments

Specific leaf area was higher in haplotype M than haplotype I2. Both haplotypes M and I2 were not different from haplotypes M1 and AI (Table 4). Chlorophyll and total carotenoid contents did not vary among and within haplotypes. However, when pigment contents were expressed on an area basis (g m^−2^) they differed significantly among haplotypes, with lower contents in haplotype M than in haplotypes I2 and M1. Haplotype AI did not differ from either haplotype M or I2 and M1 in pigment content (g m^−2^).

**Table 4:** Characteristics of four haplotypes of *Phragmites* co-existing at the Gulf Coast of North America—pigments. Mean (±1 SE) specific leaf area (SLA), chlorophyll *a* (chl*_a_*), chlorophyll *b* (chl*_b_*), chlorophyll *a*/*b* ratio (chl *a/b*), chlorophyll *ab* (chl*_a_*_+*b*_) and total carotenoid and results (*P* values) of nested two-way ANOVA. Different superscript letters in the columns indicate significant differences (*post hoc* Tukey's HSD test, *P* < 0.05) among haplotypes. Analysis of variance results in bold indicate *P* values <0.05.

Haplotype	SLA	chl*_a_*	chl*_b_*	chl *a/b*	chl*_a_*_+*b*_		Total carotenoid	
	(m^2^ kg^−1^ DM)	(mg g^−1^ DM)	(mg g^−1^ DM)		(mg g^−1^ DM)	(g m^−2^)	(mg g^−1^ DM)	(g m^−2^)
I2	17.0 ± 1.1^a^	6.23 ± 0.49	1.79 ± 0.16	3.58 ± 0.12	8.02 ± 0.64	0.474 ± 0.022^b^	1.63 ± 0.10	0.096 ± 0.004^b^
M1	19.9 ± 0.9^ab^	7.54 ± 0.42	2.07 ± 0.14	3.65 ± 0.10	9.60 ± 0.56	0.483 ± 0.019^b^	1.82 ± 0.08	0.092 ± 0.003^b^
M	24.3 ± 1.1^b^	6.57 ± 0.49	1.83 ± 0.16	3.60 ± 0.12	8.40 ± 0.64	0.351 ± 0.022^a^	1.41 ± 0.10	0.059 ± 0.004^a^
AI	23.1 ± 1.8^ab^	7.97 ± 0.84	2.35 ± 0.29	3.40 ± 0.20	10.32 ± 1.11	0.457 ± 0.038^ab^	1.73 ± 0.17	0.076 ± 0.007^ab^
Nested two-way ANOVA results (*P* value)								
Genotype (haplotype)	0.131	0.185	0.151	0.088	0.276	0.795	0.201	0.709
Haplotype	**0.018**	0.251	0.398	0.279	0.218	**0.015**	0.098	**0.001**

### Stomatal density and guard cell length

Stomatal density and guard cell length on the adaxial and abaxial sides of the leaves did not differ among haplotypes (Table 5). However, there was significant variation in adaxial stomatal density and abaxial guard cell length among genotypes within haplotypes. In general, leaves with longer stomata had lower stomatal densities.

**Table 5:** Characteristics of four haplotypes of *Phragmites* co-existing at the Gulf Coast of North America—stomatal density and guard cell length. Mean (±1 SE) stomatal density and guard cell length on the adaxial and abaxial leaf surface and results (*P* values) of nested two-way ANOVA. There are no significant differences among haplotypes (*post hoc* Tukey's HSD test, *P* < 0.05). Analysis of variance results in bold indicate *P* values <0.05.

Haplotype	Adaxial density (mm^−2^)	Abaxial density (mm^−2^)	Adaxial length (µm)	Abaxial length (µm)
I2	631 ± 42	681 ± 50	22.1 ± 0.7	22.7 ± 0.7
M1	666 ± 37	644 ± 43	20.1 ± 0.6	19.9 ± 0.6
M	664 ± 42	712 ± 50	20.2 ± 0.7	19.6 ± 0.7
AI	793 ± 74	822 ± 86	19.8 ± 1.2	20.2 ± 1.3
Nested two-way ANOVA results (*P* value)				
Genotype (haplotype)	**0.023**	0.158	0.068	**0.012**
Haplotype	0.438	0.419	0.419	0.097

### Rubisco activity

The initial Rubisco activity, the total Rubisco activity and the Rubisco activation state did not differ among haplotypes. Only total Rubisco activity differed among genotypes within haplotypes (Table 6). The activity levels (24.1–32.0 µmol C m^−2^ s^−1^ for total Rubisco activity) (Table 6) were at the same level, or slightly lower, than the light-saturated gross rates of photosynthesis, *A*_max_ (22.4–36.1 µmol CO_2_ m^−2^ s^−1^) (Table 2).

**Table 6:** Characteristics of four haplotypes of *Phragmites* co-existing at the Gulf Coast of North America—Rubisco activity. Mean (±1 SE) initial Rubsico activity (non-activated Rubisco), total Rubisco activity and Rubisco activation state (% of active Rubisco) and results (*P*-values) of nested two-way ANOVA. There are no significant differences among haplotypes (*post hoc* Tukey's HSD test, *P* < 0.05). Analysis of variance results in bold indicate *P* values <0.05.

Haplotype	Non-activated Rubisco (µmol C m^−2^ s^−1^)	Total Rubisco (µmol C m^−2^ s^−1^)	Rubisco activation state (%)
I2	14.3 ± 1.8	26.8 ± 2.8	53.9 ± 2.1
M1	15.9 ± 1.5	24.1 ± 2.4	64.2 ± 1.9
M	16.2 ± 1.8	27.4 ± 2.8	59.1 ± 2.1
AI	21.2 ± 3.2	32.0 ± 4.9	66.0 ± 3.7
Nested two-way ANOVA results (*P* value)			
Genotype (haplotype)	0.132	**0.027**	0.852
Haplotype	0.454	0.598	0.055

### Chlorophyll fluorescence

Chlorophyll fluorescence measurements revealed significant differences among genotypes within haplotypes for *F*_v_*/F*_m_ and for *I*_k,PAM_ from the rapid light-response curves (Table 7). The variability among genotypes within haplotypes was higher than the variability among haplotypes. Hence, there were no differences among haplotypes.

**Table 7:** Characteristics of four haplotypes of *Phragmites* co-existing at the Gulf Coast of North America—chlorophyll fluorescence. Mean (±1 SE) potential quantum yield of PSII (*F*_v_/*F*_m_), quantum yield (Φ_PAM_), maximum electron transport rate (ETR_max_) and light saturation point (*I*_k,PAM_) of the rapid light-response curves from chlorophyll fluorescence and the results (*P* values) of nested two-way ANOVA. There are no significant differences among haplotypes (*post hoc* Tukey's HSD test, *P* < 0.05). Analysis of variance results in bold indicate *P* values <0.05.

Haplotype	*F*_v_/*F*_m_	Φ_PAM_	ETR_max_ (µmol m^−2^ s^−1^)	*I*_k,PAM_ (µmol m^−2^ s^−1^)
I2	0.796 ± 0.005	0.319 ± 0.006	449 ± 74	142 ± 30
M1	0.788 ± 0.005	0.311 ± 0.005	599 ± 64	164 ± 26
M	0.809 ± 0.005	0.318 ± 0.006	294 ± 74	110 ± 30
AI	0.798 ± 0.009	0.285 ± 0.010	798 ± 129	227 ± 51
				
Nested two-way ANOVA results (*P* value)				
Genotype (haplotype)	**0.048**	0.131	0.191	**0.026**
Haplotype	0.136	0.079	0.055	0.267

### Principal component analysis

**Figure 3.**
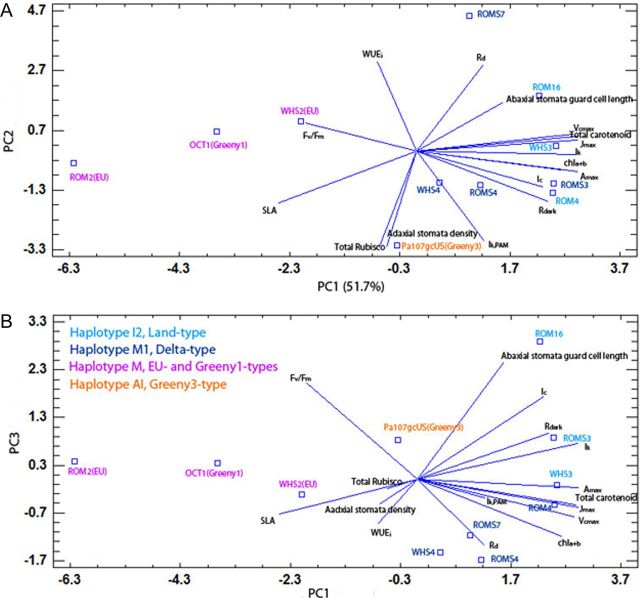
Biplots from a PCA based on all significantly different ecophysiological parameters among haplotypes and genotypes of *Phragmites* co-existing at the Gulf Coast of North America. PC1, PC2 and PC3 explain 51.7, 25.7 and 10.6 % of the variation, respectively.

Of the significantly different parameters among haplotypes and genotypes, the PCA analysis extracted three components with eigenvalues >1. Together, they accounted for 88 % of the variability in the original data. The parameters contributing the most to PC1 were *A*_max_, *V*_cmax_, *J*_max_, *I*_k_, *R*_dark_, total carotenoids and chl*_a_*_+*b*_ concentrations expressed on a leaf-area basis (all positive loadings) and SLA (negative loading). PC2 had high positive loadings from WUE_i_ and *R*_d_, and negative loadings for adaxial stomatal density, total Rubisco and *I*_k,PAM_. PC3 had high positive loadings of abaxial stomata guard cell length, *I*_c_ and *F*_v_/*F*_m_. The biplots of PC1 vs. PC2 (Fig. 3A) and PC1 vs. PC3 (Fig. 3B) separated the genotypes of the four haplotypes. Haplotypes I2 and M1 showed higher PC1 values than haplotype M (Fig. 3A). Haplotype I2 was separated from haplotype M1 along the PC3 axis (Fig. 3B). Haplotype AI was located in the lower part of the biplot (Fig. 3A), reflecting its relatively low PC2 scores. Also, ROMS7 (haplotype M1, Delta-type) was distinct from the cluster of haplotype M1's genotypes along the PC2 axis (Fig. 3A). ROMS7, unlike the other genotypes of haplotype M1, shares alleles with the EU-type genotypes of haplotype M (allele 202 at locus PaGT22; Lambertini *et al.* 2012*a*). Within haplotype M, the Greeny1-type did not appear different from the EU-type and was distinct from the Greeny3-type with haplotype AI along the PC2 axis.

## Discussion

We observed substantial differences among haplotypes and genotypes within haplotypes in the Gulf Coast of North America. Haplotypes I2 (Land-type) and M1 (Delta-type) had higher *A*_max_, *V*_cmax_, *J*_max_, *I*_k_, leaf carotenoids and chl*_a_*_+*b*_ concentrations expressed on a leaf-area basis than haplotype M (EU and Greeny1-types) (Tables 2–4). Haplotype AI had intermediate values for these parameters and was not different from either haplotype M or I2 and M1.

Genotypes varied significantly in WUE_i_, *R*_d_, adaxial stomatal density, abaxial stomatal guard cell length, total Rubisco activity, *F*_v_/*F*_m_ and *I*_k,PAM_. However, as genotypes clustered according to haplotype in the PCA when significant variation among genotypes was considered, such differences appear to be within the normal variation range within haplotypes. Only one genotype (ROMS7), probably a hybrid between haplotype M1 and haplotype M (Lambertini *et al.* 2012*a*), deviated within the cluster of haplotype M1's genotypes and showed higher WUE_i_ and mitochondrial respiration (*R*_d_), and lower *I*_k,PAM_ and adaxial stomatal density, than its close relatives within haplotype M1 and haplotype M. The geographic range in which gene flow between these two haplotypes occurred (Gulf Coast vs. Mediterranean region) is, however, still to be defined (Lambertini *et al.* 2012*a*, *b*), but the genetic and ecophysiological variation within haplotype M1 with the Delta-type phenotype is present in the Gulf Coast and deserves to be studied further in relation to the phylogenetic relationships of this group. Within haplotype M, the Greeny1-type genotype (OCT1) appears to be more similar in photosynthetic traits to the EU-type genotypes sharing haplotype M than to the genotype with haplotype AI sharing the Greeny phenotype.

As the differences among genotypes have a genetic basis—and are thus inheritable—the different haplotypes can be regarded as ecotypes. The photosynthetic characteristics of the haplotypes, their recent introduction to the Gulf Coast (Lambertini *et al.* 2012*a*), as well as the fact that they grow in direct contact (adjacent stands) in the Mississippi River Delta, suggest that the adaptations were acquired in the native, rather than in the introduced range. Nguyen *et al.* (unpubl. data) also observed the same ecophysiological differences among genotypes of the same haplotypes sampled in the native range.

The African and Mediterranean haplotypes (I2 and M1) are adapted to warmer climatic conditions than haplotype M, which has photosynthetic characteristics more similar to European *P. australis* in the temperate region (Lessmann *et al.* 2001; Hansen *et al.* 2007). Salvucci and Crafts-Brandner (2004) found similar results for plants from the desert, like *Larrea tridentata* of the southwest USA, vs. *Deschampsia antarctica*, endemic to maritime Antarctica. The other European haplotype AI had intermediate photosynthetic characteristics between the Eurasian haplotype M and the African and Mediterranean haplotypes I2 and M1. Phylogenetically, haplotype AI is closely related to a *P. australis* population in South Africa, Namibia and Botswana, a population that is introduced to Europe (Lambertini *et al.* 2012*a*, *b*).

In the present study, the *A*_max_ of haplotype M (22.4 µmol m^−2^ s^−1^) was within the range reported for *P. australis* grown in Europe (11.9–23.3 µmol m^−2^ s^−1^) (Hansen *et al.* 2007) and in the introduced range in North America (19.2–22.8 µmol) (Mozdzer and Zieman 2010). The *A*_max_ of haplotype AI (Greeny3-type) (29.1 ± 4.0 µmol m^−2^ s^−1^) was beyond the upper range of *A*_max_ values in these European populations (Hansen *et al.* 2007). The *V*_cmax_ and *J*_max_ of the four haplotypes were consistent with *A*_max_, which were higher in haplotypes I2 and M1 than in haplotype M, but not different from haplotype AI.

In our study, the *A*_max_ of haplotype M was lower than those of haplotypes I2 and M1 (Land- and Delta-types: 36.1 and 34.3 µmol m^−2^ s^−1^). In dark conditions, haplotype I2 respired more than haplotype M, as expected by the model of Prioul and Chartier (1977) for plants with higher *A*_max_. However, *R*_dark_ of haplotype M1 did not differ from that of the other haplotypes and had higher *A*_max_, *I*_c_ and *I*_k_, i.e. typical traits of a sun-adapted plant. This light response is expected for plants from warm and arid regions (Sage and Monson 1999).

The leaves of haplotype I2 were thicker (lower SLA) than those of haplotype M, and leaves of haplotypes I2 and M1 also contained higher contents of photosynthetic pigments than haplotype M, when expressed on a leaf surface area basis, probably due to more chloroplasts of smaller size in the thick leaves (Demmig-Adams and Adams 1992; Lambers *et al.* 2008). This is also consistent with sun plants (Larcher 2003). A higher number of chloroplasts in the leaves increases the number of Calvin cycles and thus the photosynthetic rates.

The studied haplotypes grow in direct contact in the Mississippi River Delta, and hence compete, but their broad distribution in the delta may also be determined by factors such as hydrological regime and salinity (Vasquez *et al.* 2005). The introduced Eurasian *P. australis* that has invaded widely in North America, and which constitutes the source population of the EU-type phenotype of haplotype M, has been studied intensively. This introduced haplotype M has been spreading and outcompeting the native *P. australis* subsp. *americanus* (Saltonstall *et al.* 2004) by means of faster growth, greater investment in sexual reproduction and more efficient seed dispersal (Howard *et al.* 2008; Belzile *et al.* 2010; McCormick *et al.* 2010, Kettenring *et al.* 2011). Furthermore, this introduced haplotype M also tolerates higher salinity levels (Vasquez *et al.* 2005), and has a greater ventilation efficiency for rhizome aeration than the native *P. australis* ssp. *americanus* (Tulbure *et al.* 2012).

Our study suggests that haplotypes I2 and M1 are superior to haplotype M in terms of photosynthetic CO_2_ assimilation. The unique genotype of haplotype AI analysed in this study lies between haplotypes I2, M1 and M. This is consistent with the current distribution of the *P. australis* haplotypes in the Mississippi Delta, with haplotype I2 (Land-type) dominating upland and haplotype M1 (Delta-type) dominating in the delta marshes. The different distributions of these two dominating haplotypes may be due to different ecophysiological characteristics not investigated in this study, e.g. differences in salinity tolerance and/or differences in the ability to aerate rhizomes or roots. Environmental differences between the delta and the uplands may also be important. Sediment accretion, variable water levels and, not least, tropical hurricanes provide windows of opportunity for the establishment of *P. australis* seedlings more frequently in the delta than in the progressively consolidated lands upstream of the delta (Cretini *et al.* 2012). Haplotypes M (both EU- and Greeny1-types) and AI (Greeny3-type) are sympatric in the delta with haplotype M1, but appear less proficient in photosynthetic CO_2_ assimilation than haplotype M1. However, these phenotypes seem to be spreading at the expense of the Land- and Delta-types (Hauber *et al.* 2011). This discrepancy might be explained by several factors. First, photosynthetic performance is only one of many factors determining the competitive ability of the *P. australis* haplotypes in the delta. Physical disturbances, water regime, salinity (Vasquez *et al.* 2005) and biotic factors such as resistance to grazing and various pests are likely to be very important (Blossey and Noetzold 1995). Secondly, our study was performed on plants in a controlled steady-state environment. In nature, the prevailing environmental conditions are changing on different temporal scales, and it might be extreme conditions, rather than the average conditions, that determine the competitive ability, and hence the fitness, of a plant (Wilcove *et al.* 1998). The origins of the four haplotypes and adaptations derived from their native climatic zones nevertheless seem to explain the differences in their photosynthetic ability.

## Conclusions

Our study has documented that four *P. australis* haplotypes co-existing in the Mississippi River Delta differ in important photosynthetic traits, and we suggest that these differences in photosynthetic characteristics are related to the climatic conditions in the native range of the haplotypes. It remains, however, to be understood how these differences, ecologically definable as ‘photosynthetic ecotypes’, relate to plant competitiveness and fitness in the Gulf Coast environment. Another aspect that deserves further attention is intra-haplotypic variation. Our study shows that hybrids might differ in ecophysiological traits—and hence in adaptive strategies—from both their parents, and that genotypes showing the same phenotype can have different origins, as well as different ecophysiological characteristics.

## Sources of Funding

This work was funded by The Danish Council for Independent Research, Natural Sciences, via a grant to H.B., and a scholarship from the Graduate School of Science and Technology, Aarhus University, Denmark, to L.X.N. Additional travel and salary support was provided by the John P. Laborde Endowed Chair for Sea Grant Research and Technology Transfer Program. The Carlsberg Foundation funded the Li-Cor 6400XT.

## Contributions by the Authors

L.X.N. conducted the experiment and drafted the manuscript. C.L., H.B., F.E., L.A. and B.K.S. participated in the experiment design. All authors read, modified and approved the final manuscript.

## Conflict of Interest Statement

None declared.

## References

[PLT016C1] Achenbach L, Lambertini C, Brix H (2012). Phenotypic traits of *Phragmites* australis clones are not related to ploidy level and distribution range. AoB PLANTS.

[PLT016C2] Armstrong J, Armstrong W (1991). A convective through-flow of gases in *Phragmites australis* (Cav.) Trin. ex Steud. Aquatic Botany.

[PLT016C3] Armstrong J, Afreen-Zobayed FB, Armstrong W (1999). *Phragmites australis*: effects of shoot submergence on seedling growth and survival and radial oxygen loss from roots. Aquatic Botany.

[PLT016C4] Belzile F, Labbe J, LeBlanc MC, Lavoie C (2010). Seeds contribute strongly to the spread of the invasive genotype of the common reed (*Phragmites australis*). Biological Invasions.

[PLT016C5] Bernacchi CJ, Pimentel C, Long SP (2003). In vivo temperature response functions of parameters required to model RuBP-limited photosynthesis. Plant, Cell and Environment.

[PLT016C6] Blossey B, Noetzold R (1995). Evolution of increased competitive ability in invasive nonindigenous plants—a hypothesis. Journal of Ecology.

[PLT016C7] Brix H (1999). On genetic diversity, ecophysiology and growth dynamics of the common reed (*Phragmites australis*). Aquatic Botany.

[PLT016C8] Brix H, Sorrell BK, Orr PT (1992). Internal pressurization and convective gas flow in some emergent freshwater macrophytes. Limnology and Oceanography.

[PLT016C9] Chambers RM, Meyerson LA, Saltonstall K (1999). Expansion of *Phragmites australis* into tidal wetlands of North America. Aquatic Botany.

[PLT016C10] Clevering OA, Lissner J (1999). Taxonomy, chromosome numbers, clonal diversity and population dynamics of *Phragmites australis*. Aquatic Botany.

[PLT016C11] Cretini KF, Visser JM, Krauss KW, Steyer GD (2012). Development and use of a floristic quality index for coastal Louisiana marshes. Environmental Monitoring and Assessment.

[PLT016C12] Demmig-Adams B, Adams WW (1992). Photoprotection and other responses of plants to high light stress. Annual Review of Plant Physiology and Plant Molecular Biology.

[PLT016C13] Dubois JJB, Fiscus EL, Booker FL, Flowers MD, Reid CD (2007). Optimizing the statistical estimation of the parameters of the Farquhar-von Caemmerer-Berry model of photosynthesis. New Phytologist.

[PLT016C14] Eller F, Brix H (2012). Different genotypes of *Phragmites australis* show distinct phenotypic plasticity in response to nutrient availability and temperature. Aquatic Botany.

[PLT016C15] Farquhar GD, Caemmerer SV, Berry JA (1980). A biochemical-model of photosynthetic CO_2_ assimilation in leaves of C-3 species. Planta.

[PLT016C16] Hansen DL, Lambertini C, Jampeetong A, Brix H (2007). Clone-specific differences in *Phragmites australis*: effects of ploidy level and geographic origin. Aquatic Botany.

[PLT016C17] Hauber DP, Saltonstall K, White DA, Hood CS (2011). Genetic variation in the common reed, *Phragmites australis*, in the Mississippi River Delta marshes: evidence for multiple introductions. Estuaries and Coasts.

[PLT016C18] Howard R, Travis S, Sikes B (2008). Rapid growth of a Eurasian haplotype of *Phragmites australis* in a restored brackish marsh in Louisiana, USA. Biological Invasions.

[PLT016C19] Kettenring KM, McCormick MK, Baron HM, Whigham DF (2011). Mechanisms of *Phragmites australis* invasion: feedbacks among genetic diversity, nutrients, and sexual reproduction. Journal of Applied Ecology.

[PLT016C20] Kühl H, Zemlin R (2000). Increasing the efficiency of reed plantations on stressed lake and river shores by using special clones of *Phragmites australis*. Wetlands Ecology and Management.

[PLT016C21] Lambers H, Chapin FS, Pons TL (2008). Plant physiological ecology.

[PLT016C22] Lambertini C, Mendelssohn IA, Gustafsson MHG, Olesen B, Riis T, Sorrell BK, Brix H (2012). Tracing the origin of Gulf Coast *Phragmites* (Poaceae): a story of long-distance dispersal and hybridization. American Journal of Botany.

[PLT016C23] Lambertini C, Sorrell BK, Riis T, Olesen B, Brix H (2012). Exploring the borders of European *Phragmites* within a cosmopolitan genus. AoB PLANTS.

[PLT016C24] Larcher W (2003). Physiological plant ecology.

[PLT016C25] Lessmann JM, Brix H, Bauer V, Clevering OA, Comín FA (2001). Effect of climatic gradients on the photosynthetic responses of four *Phragmites australis* populations. Aquatic Botany.

[PLT016C26] Lichtenthaler HK (1987). Chlorophylls and carotenoids: pigments of photosynthetic biomembranes. Methods in Enzymology.

[PLT016C27] McCormick M, Kettenring K, Baron H, Whigham D (2010). Extent and reproductive mechanisms of *Phragmites australis* spread in brackish wetlands in Chesapeake Bay, Maryland (USA). Wetlands.

[PLT016C28] Meyerson LA, Lambertini C, McCormick MK, Whigham DF (2012). Hybridization of common reed in North America? The answer is blowing in the wind. AoB PLANTS.

[PLT016C29] Mozdzer TJ, Zieman JC (2010). Ecophysiological differences between genetic lineages facilitate the invasion of non-native *Phragmites australis* in North American Atlantic coast wetlands. Journal of Ecology.

[PLT016C31] Prioul JL, Chartier P (1977). Partitioning of transfer and carboxylation components of intracellular resistance to photosynthetic CO_2_ fixation: a critical analysis of the methods used. Annals of Botany.

[PLT016C32] Rolletschek H, Rolletschek A, Kühl H, Kohl JG (1999). Clone specific differences in a *Phragmites australis* stand II. Seasonal development of morphological and physiological characteristics at the natural site and after transplantation. Aquatic Botany.

[PLT016C33] Sage RF, Monson RK (1999). C4 plant biology.

[PLT016C34] Saltonstall K (2002). Cryptic invasion by a non-native genotype of the common reed, *Phragmites australis*, into North America. Proceedings of the National Academy of Sciences of the USA.

[PLT016C35] Saltonstall K, Peterson PM, Soreng RJ (2004). Recognition of *Phragmites australis* subsp *americanus* (Poaceae: Arundinoideae) in North America: evidence from morphological and genetic analyses. SIDA Contributions to Botany.

[PLT016C36] Salvucci ME, Crafts-Brandner SJ (2004). Relationship between the heat tolerance of photosynthesis and the thermal stability of Rubisco activase in plants from contrasting thermal environments. Plant Physiology.

[PLT016C37] Sharkey TD, Bernacchi CJ, Farquhar GD, Singsaas EL (2007). Fitting photosynthetic carbon dioxide response curves for C3 leaves. Plant, Cell and Environment.

[PLT016C38] Tulbure MG, Ghioca-Robrecht DM, Johnston CA, Whigham DF (2012). Inventory and ventilation efficiency of nonnative and native *Phragmites australis* (common reed) in tidal wetlands of the Chesapeake Bay. Estuaries and Coasts.

[PLT016C39] Vasquez EA, Glenn EP, Brown JJ, Guntenspergen GR, Nelson SG (2005). Salt tolerance underlies the cryptic invasion of North American salt marshes by an introduced haplotype of the common reed *Phragmites australis* (Poaceae). Marine Ecology Progress Series.

[PLT016C40] Wilcove DS, Rothstein D, Dubow J, Phillips A, Losos E (1998). Quantifying threats to imperiled species in the United States. BioScience.

